# Dynamic metabolic and transcriptomic profiling of methyl jasmonate‐treated hairy roots reveals synthetic characters and regulators of lignan biosynthesis in *Isatis indigotica* Fort

**DOI:** 10.1111/pbi.12576

**Published:** 2016-06-23

**Authors:** Lei Zhang, Junfeng Chen, Xun Zhou, Xiaofei Chen, Qing Li, Hexin Tan, Xin Dong, Ying Xiao, Langdong Chen, Wansheng Chen

**Affiliations:** ^1^ Department of PharmacyChangzheng HospitalSecond Military Medical UniversityShanghaiChina; ^2^ Department of Pharmaceutical BotanySchool of PharmacySecond Military Medical UniversityShanghaiChina; ^3^ Analysis and Testing CenterSchool of PharmacySecond Military Medical UniversityShanghaiChina

**Keywords:** *Isatis indigotica*, lignan biosynthesis, transcriptomic profiling, metabolic profiling, co‐expression network

## Abstract

A molecular description of lignan biosynthesis in *Isatis indigotica* displaying its synthetic characteristics and regulatory mechanism is of great importance for the improvement of the production of this class of active compounds. To discover the potential key catalytic steps and regulatory genes, *I. indigotica* hairy roots elicited by methyl jasmonate (MeJA) were used as a source of systematic variation for exploring the metabolic/transcriptional changes and candidate genes that might play key roles in lignan biosynthesis. The reprogramming modulated by MeJA was classified into three distinct phases, referred to as signal responding, transcriptional activation of metabolic pathways and accumulation of metabolites. Candidate genes were pooled according to the three phases and applied to co‐expression network analysis. In total, 17 genes were identified as hub genes. *4CL3* was selected to validate its impact on lignan biosynthesis. RNAi of *4CL3* resulted in a significant reduction in lignan production. Taken together with its catalytic property, a major route of lignan biosynthesis in *I. indigotica* was highlighted, which was catalysed by 4CL3 via the esterization of caffeic acid. In conclusion, this study provides new insights into lignan biosynthesis as well as potential targets for metabolic engineering in *I. indigotica*.

## Introduction

*Isatis indigotica*, which belongs to the *Cruciferae* family, has been a widely used Chinese herb. The roots of *I. indigotica*, called *Isatidis Radix*, produce diverse chemicals with pharmaceutical properties for the clinical treatment of colds, fever and influenza (Liu *et al*., 2000; Qin *et al*., 2009; Xu *et al*., 2010). Lignans (e.g. pinoresinol, lariciresinol and lariciresinol glucosides) (Figure S1), a class of phenylpropanoid derivate, constitute one major group of effective antivirus agents in *I. indigotica* to inhibit different subtypes of human or avian influenza viruses (Li, 2003; Li *et al*., 2015; Yang *et al*., 2012, 2013). Lignans accumulate at a very low concentration (<1‰) in roots of *I. indigotica*, which impedes its clinical effect (Li, 2003). For this reason, steady progresses need to be made in understanding its biosynthesis and designing new strategies to optimize the production of these effective components in *I. indigotica*.

According to the recent transcriptome annotation of *I. indigotica*, putative lignan biosynthesis pathway in *I. indigotica* was depicted (Chen *et al*., 2013; Tang *et al*., 2014; Zhou *et al*., 2015). Biosynthesis of lignans shared a common upstream pathway with lignins and branched after the synthesis of coniferyl alcohol. A succession of specific steps, involving catalytic reaction of dirgent (DIR), pinoresinol/lariciresinol reductase (PLR), secoisolariciresinol dehydrogenase (SDH) and UDP‐glucose‐dependent glucosyltransferase (UGT), leads to the production of lignans (Figure S1). Over 50 genes were annotated to be involved in the general phenylpropanoids and lignan branch. However, it is still far away from a fully understanding of lignan biosynthesis. It is well known that multiple protein families are involved in phenylpropanoid pathways and have overlapping yet distinct roles in phenylpropanoid metabolism (Li *et al*., 2015; Staniek *et al*., 2013). There has not been a comprehensive genetic study on the impact of these isoforms on lignan metabolism. Meanwhile, no regulator relating to lignan biosynthesis in *I. indigotica* has been reported as well. Therefore, dissection of lignan metabolism is urgently needed, which allows more efficient strategies in metabolic engineering of lignan.

Plant cells generally response to MeJA induction by increasing the production of secondary metabolite. They also provide ideal models to learn the complex biochemical variation in secondary metabolism (Yan *et al*., 2014). This effect has been described in diverse medicinal plant for various classes of effective compounds. At the same time, applying high‐throughput transcriptional and metabolic analysis, candidate elements that essential for the synthetic pathways as metabolites, catalytic genes, transporters and regulators are easily captured (Kim *et al*., 2013; Luo *et al*., 2014; Pauwels *et al*., 2008; Sun *et al*., 2013; Verma *et al*., 2014).

In a previous study, we have demonstrated the effect of MeJA on *I. indigotica* hairy root cultures. Primary results showed an improvement of lignan biosynthesis pathway on both transcription and metabolic (Chen *et al*., 2013). Hence, comparing the metabolism and transcriptome of *I. indigotica* cultures elicited by MeJA with unelicited controls is a promising strategy to achieve deep insights into lignan biosynthesis. In the current study, a combination of transcriptional and metabolic profiling of MeJA‐induced *I. indigotica* hairy root was reported. Our study revealed the precious metabolic and transcriptomic tuning that was caused by perturbation of MeJA. Secondly, a regulatory network was constructed, which helped to unravel the biosynthesis genes and regulators. In addition, functional study of 4CL3 was carried out as a case study to validate the prediction made by network. These findings would facilitate a better knowledge in the mechanism of MeJA‐elicited metabolism in plant, the catalytic character and regulation of lignan in *I. indigotica*, and candidate targets for lignan engineering.

## Results

### Transcriptional profiling

To study the system‐wide changes in MeJA‐induced *I. indigotica* hairy roots, six time points (0, 1, 3, 6, 12 and 24 h after treatment) were selected for analyses. Using the paired‐end HiSeq2000 sequencing platform, 34.90–53.33 million 100‐nt reads were generated for each RNA sample (Table S1). The retained high‐quality reads were mapped to previous annotation of *I. indigotica* transcriptome (Chen *et al*., 2013). Totally, 65 196 isogenes were identified by assembly.

In each sample, 984–1583 differently expressed genes responded to MeJA were detected (compared with samples of 0 h) (Table S2). Each group shared 717 and 618 intersection of DEGs, respectively (Figure S2). The enriched GO (Gene Ontology) terms for DEGs (differently expressed genes) were analysed to evaluate the impacts of MeJA induction (Figure S3). In biology process (BP) categories, the most significant terms were ‘response to jasmonic acid’, ‘response to wounding’ and ‘jasmonic acid biosynthesis process’. The result showed JA signalling process was efficiently activated in MeJA‐induced *I. indigotica* cultures. In molecular function (MF) categories, catalytic genes (oxidoreductase activity, hydrolase activity, UDP glycosyltransferase activity, etc.) were mostly enriched. Subsequently, all DEGs were subjected to KEGG analysis to map transcriptional changes of metabolic pathways. A total of 1323 DEGs were assigned to KEGG. Genes involved in terpenoid backbone, phenylalanine, tyrosine and tryptophan synthesis were predominantly enriched (Figure S4).

### Nontargeted metabolic profiling

To have an overview of the metabolic shifts over the process in response to MeJA, nontargeted metabolic profiling was performed using UPLC‐qTOF‐MS. Seven samples collected at 0–36 h after treatment were analysed. Totally, 9254–9777 peaks were detected in each sample. To select metabolites influenced by MeJA, the statistically significant difference in the variables was tested by corresponding loading plot for the comparisons of multiple groups (Figure S5). Furthermore, the variable importance for projection (VIP) reflecting the importance of variables was applied to filter significantly changed metabolites. Metabolite ions with a VIP value >1.5 were considered as significant differential metabolites. Following the criterions above, 187 metabolites ions showed significant change in accumulation (Dataset S1). To further demonstrate how MeJA affected lignan biosynthesis, nineteen metabolites involved in lignan pathway were identified according to the accurate mass and retention time in the extracted ion chromatogram (EIC). Most of these metabolites showed significantly increased accumulation (Dataset S1).

### Systematic transcriptome and metabolites shifts modulated by MeJA in *Isatis indigotica* hairy roots

**Figure 1:**
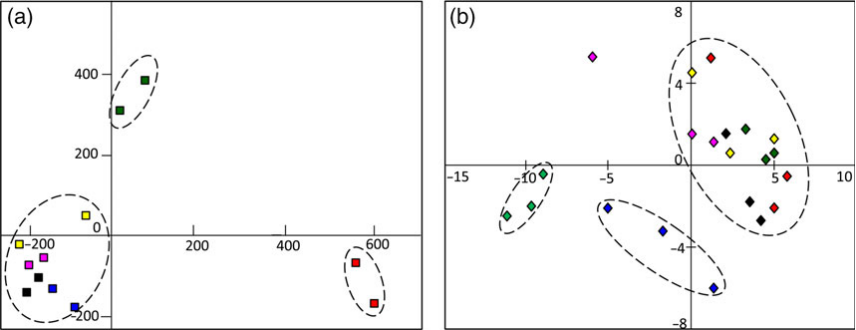
Transcriptomic and metabolic shifts modulated by MeJA in *I. indigotica* hairy roots. (a) PCA reveals transcriptomic shifts during a time course of 0–24 h. (b) PCA reveals metabolic shifts during a time course of 0–36 h. Plots with different colours indicate samples of different time points. Plots in red indicate control groups (0 h). Dark green plots, yellow plots, pink plots, black plots, blue plots and green plots indicated samples treated by MeJA for 1, 3, 6, 12, 24 and 36 h, respectively.

Principal component analysis (PCA) was employed to display the dynamic variation over the MeJA response course. As shown in Figure 1a, plots representing overall transcripts from individual samples of 0 and 1 h showed a distinct sample separation. Plots representing samples of 3–24 h clustered well and separated to samples of 0 and 1 h. The results indicated two different transcriptional variation tendencies that represented in the 1 h and following stage (3–24 h). In other words, all DEGs could be generally classified into two classes as early stage and late stage of MeJA‐responsive genes (Pauwels *et al*., 2008).

Compare to transcriptome, metabolic variation showed different tendencies by PCA profile. As a result of mass spectrometry detection in the positive ion mode, the plots representing samples of 0–12 h were clustered (Figure 1b). However, plots representing samples of 24 and 36 h showed distinct separations, suggesting significant metabolic variations in these samples. Similar results were also presented in the negative ion mode (Figure S6). Evidently, the majority of metabolic variation was detected at late stage over the course, which was in an opposite tendency to that of transcriptome. These results presented a time gap from transcriptional variation to the accumulation of metabolites over the response course to MeJA (Shulaev *et al*., 2008).

### Transcriptomic and metabolic profile of lignan metabolism modulated by MeJA

**Figure 2:**
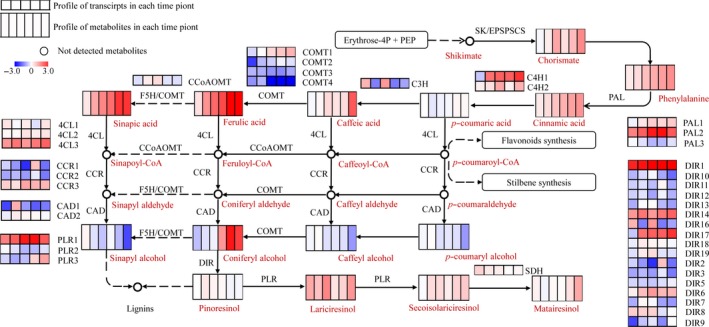
Transcriptomic and metabolic shifts in lignan metabolism as a result of MeJA induction. Heat maps show transcriptional and metabolic shift in samples of each time point. Dashed arrows indicate conversion catalysed by multiple steps. 4CL, 4‐coumarate: CoA ligase; C4H, cinnamate 4‐hydroxylase; CAD, cinnamyl alcohol dehydrogenase; CCoAoMT, caffeoyl‐CoA *O*‐methyltransferase; CCR, cinnamoyl‐CoA reductase; COMT, caffeic acid 3‐*O*‐methyltransferase; CS, chorismate synthase; DIR, dirgent; EPSPS, 5‐enolpyruvylshikimate‐3‐phosphate synthase; F5H, ferulate‐5‐hydroxylase; HCT, p‐hydroxycinnamoyl‐CoA shikimate quinate hydroxycinnamoyl transferase; PAL, phenylalanine ammonia‐lyase; PLR, pinoresinol/lariciresinol reductase; SDH, secoisolariciresinol dehydrogenase; SK, shikimate kinase.

Metabolic analysis revealed that the production of lignans was significantly increased by MeJA induction. To have a systematic view on the variation of lignan biosynthesis pathway, we observed abundances of 58 transcripts coding 16 catalytic genes and 15 metabolites involved in lignan biosynthesis. The entire pathway could be divided into synthesis of phenylalanine, general phenylpropanoid pathway and lignan specific pathway (Figure 2). Most identified metabolites showed a significantly improved production in at least one time point. Accumulation of chorismate, which was the common precursor of tryptophan (Trp), tyrosine (Tyr) and phenylalanine (Phe) in primary metabolism, was significantly increased by MeJA. Correspondingly, Trp, Tyr and Phe showed an improved accumulation (Dataset S2). The results confirmed that the MeJA‐modulated activation of phenylpropanoid pathway was initiated with the primary metabolites (Yan *et al*., 2014). Following this, most metabolites, which are involved in general phenylpropanoid pathway (e.g. cinnamic acid, *p*‐coumaric acid, caffeic acid, ferulic acid and sinapic acid.), and lignan compounds were markedly increased.

Diverse transcriptional profiles of isoforms coding different gene families were observed. For instance, various catalytic genes (e.g. *PAL2*,* C4H1*,* 4CL3* and *PLR3*) showed similar up‐regulated temporal patterns in correspondence with increased metabolites, suggesting their roles associated with increase in metabolites.

### Temporal patterns of gene expression in MeJA‐treated *Isatis indigotica* hairy roots

To have insights on transcriptomic shifts modulated by MeJA, all DEGs over MeJA induction course were grouped in clusters according to their temporal profiles using series cluster. As PCA analysis indicated, samples of 6, 12 and 24 h represented similar transcriptional characteristics (Figure 1a). Therefore, these three time points were combined for simplicity. Samples of 0, 1 and 3 h were analysed as individual time points.

**Figure 3:**
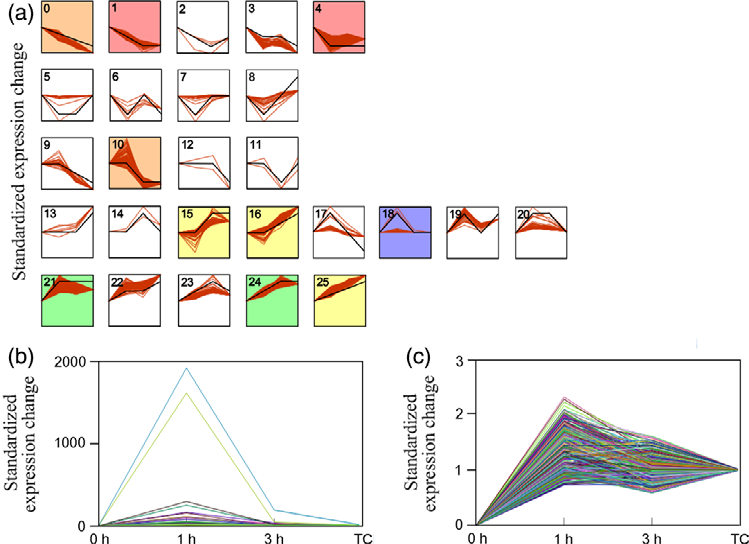
Temporal patterns of different expressed genes in response to MeJA induction. (a) 26 expression patterns of all DEGs. Gene expression profiles of individual genes are depicted in red lines, and average expression profiles for each series are depicted in black lines. Each cluster is numbered according to their expression patterns. Line charts with background colour indicate significant expression difference of the cluster. (b) Cluster 18 represents the profile of 418 transcripts that are primarily involved in JA signalling. (c) Cluster 21 represents the profile of 341 transcripts that are primarily related to metabolic pathways.

Totally, 26 expression patterns of 2562 DEGs were grouped (Figure 3a), with ten of which showed significant transcriptional changes with false discovery rate (FDR) value >0.5 (Dataset S2). The largest cluster (cluster 4) was made of 759 one‐step down‐regulated (transcription‐level transitions from high to low in two consecutive stages) genes. GO analysis showed that transcriptional regulators were enriched in MF categories. Few metabolic genes were shown in this cluster (Figure S7).

To understand the nature of regulatory processes of MeJA induction, we focused on two up‐regulated clusters (18 and 21) according to GO distribution analysis of all series clusters. The two clusters covered the majority of DEGs involved in reprogramming of metabolism and JA signalling. Cluster 18 enriched the most up‐regulated genes (Figure 3b) with 418 genes showed two‐step up/down‐regulation (transcription‐level transitions from low to high and back down in series stages). Major GO terms assembled in this cluster were ‘response to jasmonic acid’ and ‘jasmonic acid biosynthetic process’ of BP category, ‘allene oxide cyclase activity’ and ‘regulation of transcription’ of MF category (Table 1). These GO terms mainly included genes involved in JA synthesis, JA signalling and potential relative regulators. These data indicated gene expression programmes in early MeJA signalling process. Cluster 21 showed enrichment of 341 one‐step up‐regulated DEGs (Figure 3c). Transcriptional up‐regulation was observed at 1 h and maintained until the later stages. GO classifications mainly presented terms of metabolic pathways (e.g. indole acetic acid biosynthesis and tryptophan catabolic process) and catalytic activity (Table 1), implying an enrichment of metabolic genes positively regulated by MeJA.

**Table 1:** GO categories show the most significant biological process and molecular function terms falling into clusters 18 and 21

Cluster	Biological process (BP)			Molecular function (MF)		
	GO terms	Number of DEGs	*P*‐value	GO terms	Number of DEGs	*P*‐value
Cluster 18	Response to jasmonic acid	46	5.74E‐24	Allene oxide cyclase activity	3	2.83E‐04
	Jasmonic acid biosynthesis process	36	5.74E‐24	Transcription factor activity	45	1.28E‐03
	Response to wounding	50	3.01E‐23	Transcription regulatory DNA binding	3	2.10E‐03
	Response to fungus	28	3.78E‐18	Dioxygenase activity	6	2.27E‐03
	Jasmonic acid‐mediated signalling	35	4.67E‐14	Acyl‐CoA hydrolase activity	3	2.55E‐03
	Transcription factor activity	12	2.66E‐08	IAA‐amino acid conjugate hydrolase	2	2.97E‐03
Cluster 21	Indoleacetic acid biosynthetic process	20	3.68E‐15	Catalytic activity	43	1.71E‐05
	Tryptophan catabolic process	18	6.4E‐15	Oxidoreductase activity	35	1.73E‐04
	Cellular amino acid biosynthetic process	21	5.71E‐12	Haem binding	14	2.21E‐04
	Jasmonic acid biosynthetic process	16	3.22E‐09	Oxidoreductase activity,	11	3.37E‐04
	Response to jasmonic acid	21	7.66E‐09	Oxygen binding	10	3.39E‐04
	Tryptophan biosynthetic process	7	5.29E‐08	Pyridoxal phosphate binding	8	7.73E‐04

KEGG mapping of pathway was carried out to further analyse gene composition in cluster 21. Eighty‐two genes were mapped to pathway annotation (Dataset S2). Diverse primary metabolic pathways (e.g. phenylalanine, tyrosine and tryptophan biosynthesis) and secondary metabolic pathways (e.g. phenylpropanoid biosynthesis, stilbenoid, isoquinoline alkaloid biosynthesis, indole alkaloid biosynthesis and terpenoid backbone biosynthesis) fell in this cluster. DEGs with one‐step up‐regulation also fell in other clusters, but without a significant enrichment, such as clusters 15, 16, 24 and 25 (Figure 3a).

### Co‐expression network of MeJA‐mediated transcriptional reprogramming

As a first step towards understanding reprogramming of secondary metabolic in *I. indigotica*, the temporal patterns of DEGs provided distinct pools of candidate genes involved in signalling and synthesis. However, the nature of the relationships between the various metabolic genes and their regulatory factors was remained to be determined. To further explore the regulation mechanism mediated by MeJA, a co‐expression network based on Pearson correlation coefficient of gene pairs over multiple time points was constructed. In such a network, transcripts (nodes) are linked (edges) to perform the correlation of abundance and further indicate their correlated *in vivo* roles (Saito *et al*., 2008; Saito and Matsuda, 2010).

We firstly constructed a correlation network based on 410 DEGs mainly selected from clusters 18 and 21 (Figure 3). In addition, different expressed TF genes from different families were also screened (Dataset S3). The correlated pairs were filtered by correlation coefficient (>0.99). The visualization in Cytoscape (Shannon *et al*., 2003) revealed that a total of 345 nodes were connected in the network with 2748 edges (Figure S8, Dataset S4). One thousand eight hundred and two pairs of genes showed a positive correlation and 1032 pairs were negatively correlated. The weight of each node was expressed as k‐core value (1–16). Eighty‐one nodes (46 metabolic genes and 35 TF genes) presented the highest k‐core value of 16, implying their core roles in the network.

**Figure 4:**
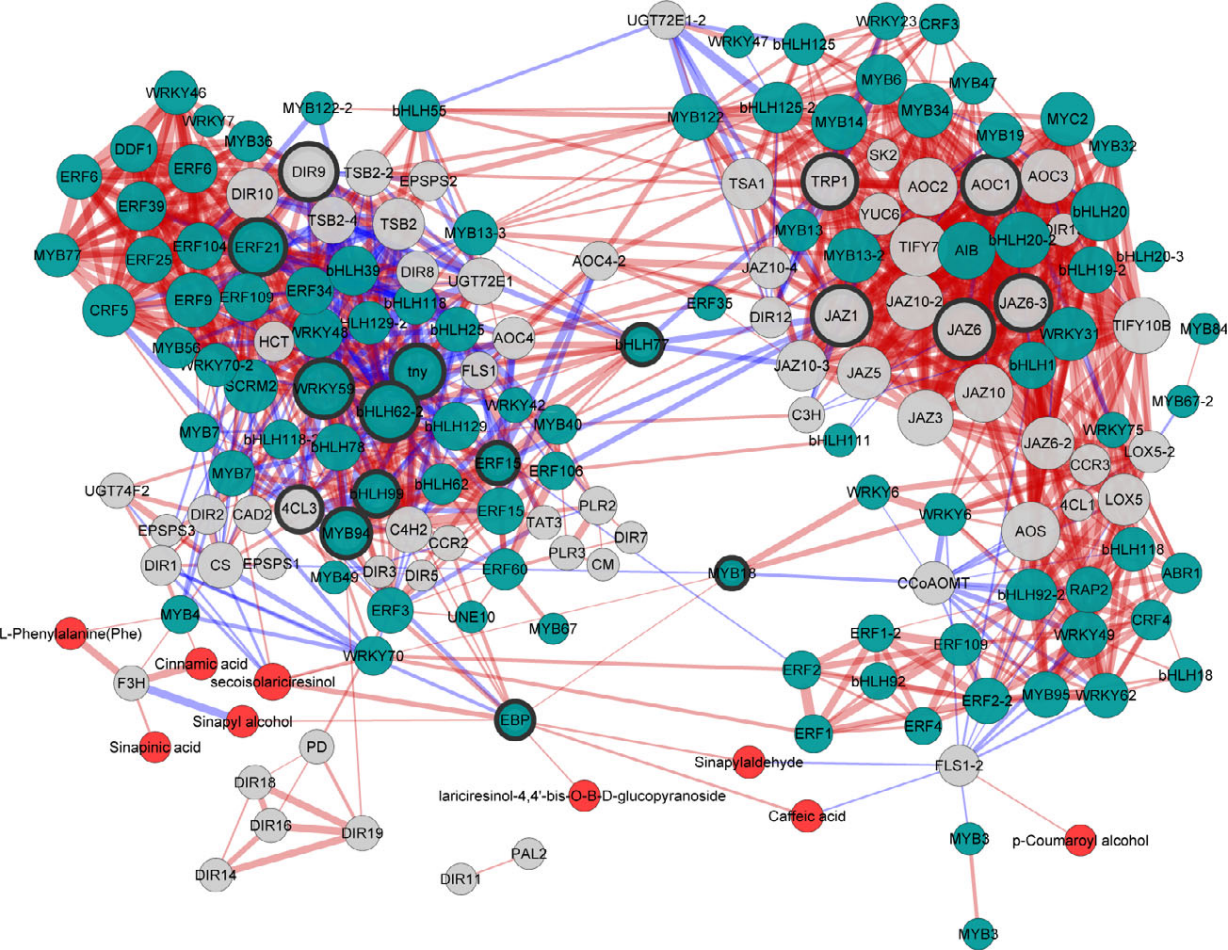
The Pearson correlation network reveals lignan synthesis and regulation. Different colour of nodes presents TF genes (green), metabolic genes (grey) and metabolites (red). Size of each node indicates amount of correlated genes. Red edges represent positive correlations and blue edges represent negative correlations. Thickness of each edge represents the value of correlation coefficient for each correlated pairs. Hub genes are highlighted with thick edging.

To further model synthetic and regulatory characters of lignan biosynthesis, a subnetwork representing transcript–metabolite correlation was constructed. The transcripts involved in shikimate and phenylalanine synthesis, the general phenylpropanoid pathway, lignan biosynthesis and metabolites in whole pathway were subjected to correlation test. A transcript–metabolite correlation network was built that consisted of 175 nodes and 1956 edges (Dataset S5). As shown in Figure 4, the visualized network was divided into two distinct subnetworks. Components associated with JA signalling mainly fell into one cluster, including several core JA signalling components as AOC1, MYC2 and JAZs (Fernández‐Calvo *et al*., 2011; Kazan and Mannaers, 2013). Another cluster mainly consisted of synthetic genes, metabolites and correlated TFs, indicating their ‘gene–metabolite’ correlation in synthesis and regulation processes (Higashi and Saito, 2013).

### Putative regulators in lignan biosynthesis

Hub genes presented to play a central regulatory role in the network. In the network constructed, ten TF genes were represented as hub genes and fell into two subclusters (Table 2), indicating their essential roles in transcriptional regulation. According to functional annotations of their reference genes, six candidates were involved in hormone response, phytochrome signalling and stress response. Meanwhile, four candidate TFs had no functional annotations. To be noted, two hub regulators (bHLH77 and MYB18) connected two subclusters with multiple correlations to other TF genes or synthetic genes, implying their specific importance in the network.

**Table 2:** Transcriptional factors predicted to act as key regulators of lignan synthesis in *Isatis indigotica*

Gene name	Reference gene name	Functional annotation of reference gene
ERF15	AT2G31230	ABA response
ERF21	OsAP21	ABA response
WRKY59	AT2G21900	ABA response
bHLH62‐2	AT3G07340	None
bHLH77	AT3G23690	Auxin response
bHLH99	AT5G65320	None
MYB18	AT4G25560	Phytochrome A signalling
MYB94	AT3G47600	Wax biosynthesis
TNY	AT5G25810	None
EBP	AT3G16770	Oxidative and osmotic stress responses

### 4CL3 plays distinct role in lignan biosynthesis

Besides regulators, co‐expression networks also provided candidate genes in particular synthetic pathways (Goossens, 2015). *4CL3* and *DIR9* were the only two synthetic genes presented as hub genes. *DIR9* showed a negative correlation to other elements in the network. Hence, we selected 4CL3 to validate its putative key role. 4CLs catalyse the first branch point of the general phenylpropanoid pathway, which leads phenylpropanoid metabolism to different branch pathways due to specific amino acid residues in the substrate binding pocket (Schneider *et al*., 2003). Therefore, we selected 4CL3 to validate its putative key role by comparative functional study of three 4CL family proteins identified via transcriptome annotation of *I. indigotica*.

Expression pattern of 4CL family was examined firstly. To investigate the subcellular localization of 4CLs, each *4CL* gene was *C*‐terminal fused to the green fluorescent protein (GFP). Confocal microscopy analysis showed all 4CL‐GFP fusion proteins had a cytosolic expression pattern (Figure S9a). Meanwhile, three *4CL* genes showed higher transcription level in roots compared to that of stems and leaves (Figure S9b), indicating similar organs expression specificity. Then, *in vitro* catalytic characterization of 4CLs using recombinant protein expressed in *Escherichia coli* was performed (Figure S10). 4CLs catalyse conversion of hydroxycinnamic acids to their corresponding coenzyme A (CoA) esters, but with different substrate preference (Schneider *et al*., 2003). We used cinnamic acid, *p*‐coumaric acid, caffeic acid, ferulic acid and sinapic acid as substrates. According to values of *K*_m_ and *V*_max_ (Table 3), 4CL1 did not have catalytic activities to any of the tested substrates. 4CL2 catalysed conversion of *p*‐coumaric acid, caffeic acid, ferulic acid and sinapic acid, and showed a high affinity to caffeic acid and ferulic acid with apparent *K*_m_ of 8 ± 0.70 and 6 ± 0.90, respectively. 4CL3 presented conversion activities to cinnamic acid, caffeic acid and ferulic acid. The highest affinity towards caffeic acid (*K*_m_ *= *3 ± 0.40) was observed. As above, enzyme activity demonstrated the different catalytic property of each 4CL.

**Table 3:**
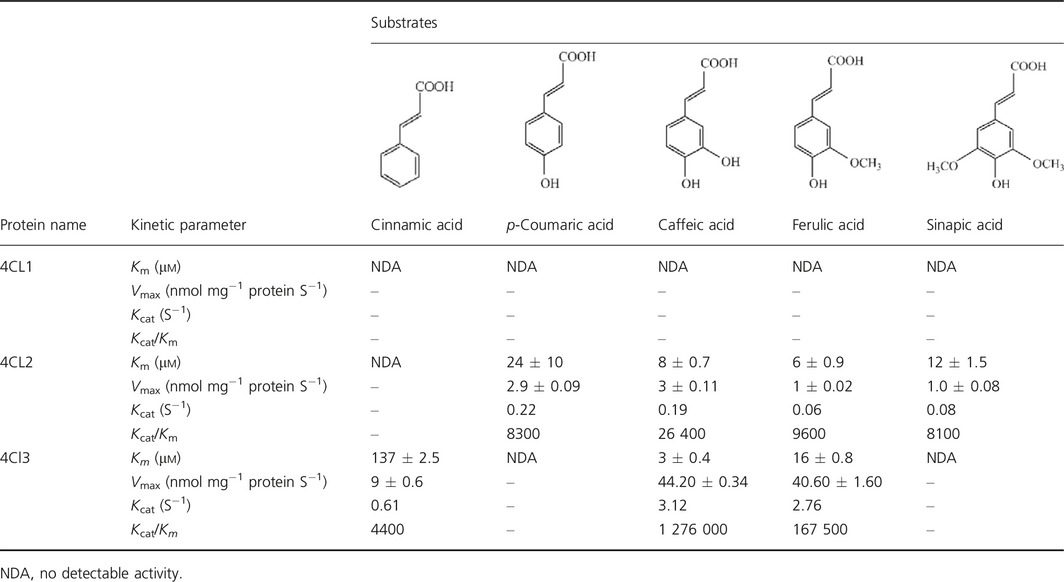
Kinetic constants of three 4CLs with various hydroxycinnamic acid substrates

To further demonstrate contributions of each 4CL to lignan biosynthesis, we constructed RNAi lines *4CL* gene to investigate the effect of each 4CL on lignan biosynthesis. Expression level of each *4CL* gene was detected in established RNAi lines firstly.

**Figure 5:**
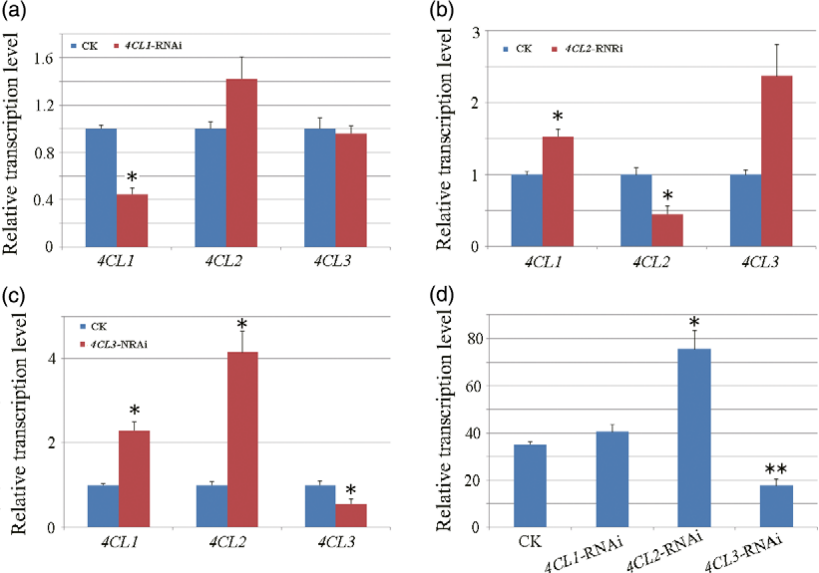
Phenotyping of *4CL* expression reduced RNAi hairy roots. qRT‐PCR detected transcription level of *4CL1*,* 4CL2* and *4CL3* in RNAi‐transgenic hairy roots of (a) *4CL1*, (b) *4CL2* and (c) *4CL3* compared with that of controls (empty vector transgenic lines). (d) Content of lariciresinol in transgenic hairy roots. All transcripts and metabolites were detected for three biological replicates. Asterisks indicate statistically significant differences compared with control (Student's t‐test, *P <* *0.05, **P <* *0.01).

qRT‐PCR analysis showed that the transcript level of the target gene (*4CL1*,* 4CL2* and *4CL3*) was significantly suppressed through corresponding RNAi manipulation, with the transcript level of the other two *4CL* members displaying a slight or significant increase (Figure 5a–c). To further demonstrate the different impacts of each 4CL on lignan biosynthesis, accumulation of lariciresinol was detected (using LC‐MS/MS method) in comparison with control lines. As shown in Figure 5d, lariciresinol level had no obvious change in *4CL1*‐RNAi lines, increased significantly (1.89‐fold in average, *P*‐value < 0.05) in *4CL2*‐RNAi lines and reduced significantly (49.3% in average, *P*‐value < 0.01) in *4CL3*‐RNAi lines. Obviously, only 4CL3 showed a positive correlation to lariciresinol production, suggesting its unique role in lignan biosynthesis.

**Figure 6:**
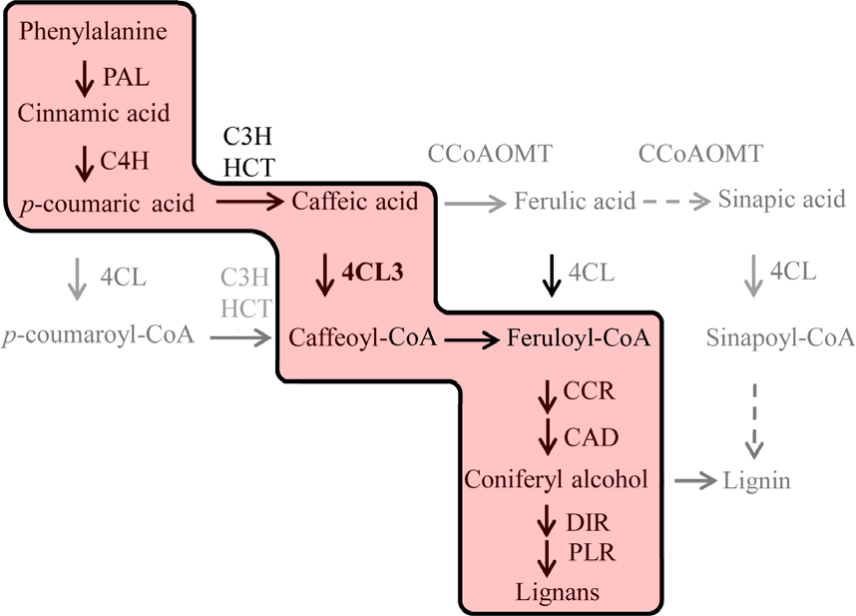
Specific synthesis route of lignan in *I. indigotica*. The putative route mainly leading to synthesis of lignans is highlighted by red background. Full names of the genes are given in Figure 2.

It is well known that 4CL family proteins catalyse the conversion of CoA esters of a series of hydroxycinnamic acid in complex phenylpropanoid metabolism (Dixon and Reddy, 2003; Li *et al*., 2015; Sun *et al*., 2013; Zhao and Dixon, 2011). Coupling the *in vitro* enzyme kinetics with *in vivo* functional features, our result showed that 4CL3 acquired a distinct role in lignan metabolism in *I. indigotica,* which was dominatingly via the conversion of caffeic acid catalysed by 4CL3 (Figure 6).

## Discussion

MeJA elicitation has a general promotion effect on accumulations of a wide range of valuable metabolites in plants as terpenoids, phenylpropanoids and alkaloids (Luo *et al*., 2014; Sun *et al*., 2013; Verma *et al*., 2014). Meanwhile, different metabolites may respond to elicitation in diverse patterns and with unique species‐specific manners. Therefore, in this study, we dissected the MeJA‐responsive molecules during the entire time course to study lignan biosynthesis in *I. indigotica*. The results demonstrated the precise biological processes to show the metabolic variation and uncovered candidate genes that play important roles for the accumulation of metabolites.

The biological processes after MeJA elicitation integrate in a complex manner to control lignan synthesis whereby underling genes be involved in signalling and synthetic processes. For simplicity, our results classified the majority of MeJA‐responsive process into three clearly distinguishable stages or aspects. The first stage reflected events that were involved in the JA signalling pathway. Transcripts falling into this group showed dramatically up‐and‐down temporal pattern (Figure 3). Most representative events were genes encoding the JAZ/MYC2 cascade (e.g. *MYC2*,* JAZ6* and *TIFY10*) that interplayed the reserved central role of JA signalling (Chini *et al*., 2007; Yan *et al*., 2007). Meanwhile, synthetic genes of jasmonate (e.g. *AOC2*,* AOS* and *LOX3*) and multiple TF genes cooperated with the central JAZ/MYC2 cascade in a coordinated pattern and might execute fine‐tuning control of a downstream gene expression (Pauwels and Goossens, 2008). The majority of the second group was mainly made up of genes involved in metabolic pathways that showed continuous one‐step up‐regulation. In the third stage (12 h or later after elicitation), transcriptome did not show notable variations. On the contrast, major metabolic variations were detected at this period. Meanwhile, it presented a time lag effect of metabolism variation as cumulative effects of transcriptional variation (Shulaev *et al*., 2008). Taken together, the division of three stages helped to classify the underlying genes and metabolites that involved in the reprogramming modulated by MeJA.

The phenylpropanoid pathways in *I. indigotica* were divided into different branches and subjected to MeJA elicitation in different patterns. This study showed that most genes and metabolites known to be involved in lignan biosynthesis were more highly expressed after MeJA elicitation (Figure 2). Nineteen metabolites involved in lignan pathway were identified, and ten of them were significantly increased (log2FC > 1, compared to mock) in at least one of the sampled period during the observed time course. This result, together with the activated tricarboxylic acid (TCA) cycle and shikimate synthesis observed in Arabidopsis (Pauwels *et al*., 2008; Yan *et al*., 2014), strongly suggested the role of MeJA as universal elicitor for both primary and secondary metabolites (Hanik *et al*., 2010). However, not all metabolic pathways were regulated by MeJA. For instance, the indole metabolism, another major class of effective compounds in *I. indigotica*, showed slight perturbations in response to MeJA elicitation. This fact was not consistent with previous observation in Arabidopsis, in which indole metabolism was decreased by JA signalling (Hanik *et al*., 2010; Kim *et al*., 2015) due to the interaction of IAA/JA signalling (Gutierrez *et al*., 2012). This result is also an evidence for in the species‐specific metabolites regulation of MeJA.

Another aspect of lignan biosynthesis in *I. indigotica* that needs to be focused is to uncover the key catalytic enzymes and regulators that determined the production of lignan. For this purpose, we generated a gene–metabolite network including both transcripts and metabolites to model this specific metabolic pathway. The topology of this network presented two distinguished subclusters (Figure 4), which presented the correlation of signalling and metabolic modules, respectively. The result also consisted of temporal analysis that signalling events and metabolic pathways presented different temporal patterns in response to MeJA. Although the current network could not fully display the synthetic and regulatory mechanism of lignan biosynthesis, it effectively provided information for prediction of key enzymes. In the network, two synthetic genes (*4CL3* and *DIR9*) were presented as hub genes, suggesting their key roles as rate‐limiting steps. We validated the role of 4CL3 in comparison with other two 4CL proteins. The *in vivo* functional study demonstrated highly significant effects of 4CL3 on the accumulation of lignans. To be further taken together with their substrate preference, a distinguishable route that was specific for lignan synthesis could be speculated. (Figure 6). Interestingly, 4CL1 showed no catalytic activity to any tested hydroxycinnamate, which represented obvious separation to other plant 4CLs by phylogenetic analysis (Figure S11). The loss of function may be due to multiple mutations of conserved residues that essential for substrate binding (Figure S12) (Gao *et al*., 2015; Hu *et al*., 2010). In previous study, we have demonstrated the dominant role of pinoresinol/lariciresinol reductase 1(PLR1) in lignan synthesis in *I. indigotica* (Xiao *et al*., 2015), whereas the precise role of protein families (e.g. PAL, CCR, CAD and DIR) that involved in the rest catalytic steps of lignan biosynthesis still remains as areas of intensive study. Typical of most enzymes involved in phenylpropanoid pathways are found to be encoded by multiple genes and supposed to contribute to independent branches for biosynthesis or distinct metabolic functions (Dixon and Reddy, 2003). A recent report described functional and structural analyses for isoforms of CCR and CAD from *Medicago truncatula* in synthesis of phenylpropenyl alcohols (Pan *et al*., 2014). Among these alcohols, coniferyl alcohol is the common precursor of both lignan and G‐lignin (Figure 2). Thus, this finding may be of reference meaning for the understanding of lignan biosynthesis.

An important function of co‐expression network is to discover new genes encoding both catalytic enzymes and regulators in metabolic pathways. For instance, glucosylation of lignans may be an important conversion step for their biological activity. Nevertheless, the corresponding catalytic UGTs are not identified yet. To identify corresponding *UGT* genes, all UGT‐encoding genes form *I. indigotica* transcriptome were valued for network construction. As a result, only two of them (UGT74F2 and UGT72E1‐2) were presented in the network, indicating their glycosylation activity towards lignans. A phylogenetic analysis further supported this prediction (data not shown) for closest evolutionary distance between UGT74F2 and a known UGT (flax UGT74S1) with catalytic property towards lignan (Ghose *et al*., 2014; Ghose *et al*., 2015). In general, most hub genes in a co‐expression network are TF genes, indicating their core regulatory role in the network (Babu *et al*., 2004). Totally, 10 TF genes presented as hub genes in our network. Some of these TFs were annotated against known genes with detailed function studies as ERF15 (Çevik *et al*., 2012) and MYB94 (Lee and Suh, 2014). On the contrary, most of the predicted core regulators were noninformative genes. Therefore, the further challenge will be to discover the precise role of these TFs in controlling lignan synthesis. More importantly, these genes can also be considered as rationally engineered targets that leading to high‐level production of lignan in transgenic plants.

In conclusion, this study has strategically used MeJA‐elicited cultures to provide information on transcriptome and metabolic variation in *I. indigotica*. The profiling coupled with transcriptome and untargeted/targeted metabolite analyses are good examples for understanding lignan biosynthesis in a species‐specific manner. Whereas hairy root cultures were used as plant cell systems in this study, our results may not fully reflect the transcriptional and metabolic traits in *I. indigotica* plants. For instance, biological variations with organ specificities or in certain developmental stages may not happen in hairy roots. Therefore, the current study establishes good theoretical basis and methods to further ascertain accumulation pattern of lignan in a whole *I. indigotica* plant within an entire growth cycle and corresponding mechanism, and finally will lead to a complete understanding for biosynthesis of lignan in this medicinal plant.

## Experimental procedures

### Plant materials and treatment

*Isatis indigotica* hairy root cultures were maintained and subcultured in our laboratory. The hairy root material was cultured in 200 mL of 1/2 B5 liquid medium at pH 5.6. After 3–4 weeks of shaking culture in dark at 25 °C, the cultures at the exponential phase were prepared for induction. A sample of 0.5 μm of MeJA (Sigma‐Aldrich, St. Louis, MO) dissolved in ethanol was added to 200 mL of 1/2 B5 liquid medium for the induction. Solvent at the same volume was added into the control group. Hairy root cultures were collected at 0, 1, 3, 6, 12, 24 and 36 h after treatment. Each biological sample was collected individually for following metabolic and transcriptional profiling and frozen immediately in liquid nitrogen for storage at −80 °C.

The plant of *I. indigotica* was grown in the medicinal plant garden of the Second Military Medical University, Shanghai, China. Flowers, leaves, stems and roots of flowering plantlets were collected. Organs were frozen immediately in liquid nitrogen and storage at −80 °C for RNA isolation.

### RNA isolation and transcriptome sequencing

Total RNA was isolated with TRIzol reagent (Tiangen, Beijing, China) according to the protocol of the manufacturer. For each sample, mRNA was purified using oligo (dT)‐attached beads and fragmented into small pieces (100–400 bp). cDNA libraries were constructed using TruseqTM RNA sample prep Kit (Illumina, San Diego, CA). Sequencing was performed on an Illumina HiSeq2000 platform (Illumina). Totally, 12 samples (six time points × two biological) were sequenced. After sequencing, the raw reads were generated using Solexa GA pipeline 1.6. After the removal of low‐quality reads, processed reads with an identity value of 95% and a coverage length of 100 bp were assembled using the Trinity *de novo* assembler (http://trinityrnaseq.sourceforge.net/).

The raw RNA‐seq read data are deposited in the Short Read Archive (http://www.ncbi.nlm.nih.gov/sra/) and are accessible through accession number SRP053268.

### Untargeted metabolic profiling

Chemical extraction was carried out on 18 samples (seven time points × three biological) for metabolic analyses. Samples were freeze‐dried and powdered for extraction. One hundred milligram of each sample was presoaked with methanol and then extracted with methanol under sonication for 30 min for three times. The extraction was evaporation‐dried and redissolved in 100 μL methanol for analysis.

Chemical analysis was performed using an ultra‐performance liquid chromatography system (UPLC, Agilent 1290; Agilent Technologies, Waldbronn, Germany) fitted with an Agilent 6538 UHD Accurate‐Mass Q‐TOF LC/MS (MS‐TOF; Agilent Technologies, Santa Clara, CA) equipped with an ESI interface. The chromatographic separation of compounds was achieved using an Agilent Eclipse Plus C18 column (2.1 × 100 mm, 1.8 μm) in binary gradient mode at a flow rate of 0.3 mL min^−1^. Column oven and autosampler temperatures were maintained at 25 and 4 °C, respectively. The sample injection volume was 5 μL. The full‐scan mass spectra were measured in a scan range from 100 to 1500 amu with a scan resolution of 13 000 m/z/s. Spectra were acquired in the positive and negative ionization modes. Data analysis was performed using Agilent Mass Hunter Workstation software. Identification of target compounds was carried out according to their corresponding quasi‐molecular ion peak. First, the corresponding quasi‐molecular ion peak was found according to the retention time in the extracted ion chromatogram (EIC). Second, the element composition of the peak was calculated by Agilent MassHunter software according to the extract mass and isotope pattern. Third, the elemental composition and mass fragmentation were compared to those registered in accessible databases of HMDB (http://www.hmdb.ca), METLIN (http://metlin.scrippps.edu) and KEGG (http://kegg.jp). In this way, 34 metabolites were identified.

### Statistical analysis

The data of transcriptome and metabolites profiling were normalized and exported to SIMCA‐P V12.0.0 Demo (Umetric, Umea, Sweden) for principal components analysis (PCA) and partial least‐squares discriminant analysis (PLS‐DA). One‐way ANOVA was performed to reveal the statistical differences in the significance of variation among each sample during the time course. The significance of differences between the groups was evaluated by the *P*‐value for the fixed‐effect parameter estimate of group differences. Variables that significantly contributed to the clustering and discrimination were identified according to a threshold of variable importance in the projection (VIP) values, which could be generated after PLS‐DA processing.

We applied DEseq algorithm to filter the differentially expressed genes according to their reads per kilobase of exon model per million mapped reads (RPKM). After significant analyses and FDR analyses under the following criteria, (i) fold change > 2 or <0.5 and (ii) FDR < 0.05 (Anders and Huber, 2010).

Gene Ontology (GO) analysis was performed to facilitate elucidating the biological implications of unique genes in the significant or representative profiles of the differentially expressed gene in the experiment (Ashburner *et al*., 2000). GO annotations were downloaded from NCBI (http://www.ncbi.nlm.nih.gov/), UniProt (http://www.uniprot.org/) and the Gene Ontology (http://www.geneontology.org/). Pathway analysis was used to find out the significant pathway of the differential genes according to KEGG database (Ashburner *et al*., 2007). We turned to the Fisher's exact test to select the GO categories and significant pathway, and the threshold of significance was defined by *P*‐value and FDR (Draghici *et al*., 2007).

Before temporal pattern analysis, differentially expressed genes were selected between samples of 0, 1, 3 h and the combination of *t* 6, 12 and 24 h. In accordance with different RPKM change tendency of genes, a set of unique model expression tendencies were identified. The expression model profiles are related to the actual or the expected number of genes assigned to each model profile. Significant profiles had higher probability than expected by Fisher's exact test and multiple comparison test (Ramoni *et al*., 2002).

Co‐expression networks were presented to find the relations among genes (Pujana *et al*., 2007). Networks were built according to the normalized expression values of genes selected from genes in significant GO terms. For each pair of genes, the Pearson correlation was calculated. Significant correlation pairs (FDR < 0.05) were chosen to construct the network. Within the network, relative importance of each gene is determined by degree centrality. Degree centrality is defined as the link numbers of one node. Moreover, to study some properties of the networks, k‐cores in graph theory are introduced as a method of simplifying graph topology analysis. A k‐core of a network is a subnetwork in which all nodes are connected to at least k other genes in the subnetwork. The hub genes among the network were identified according to topological coefficient of each node with degree > 30 or betweenness centrality > 0.05 or closeness centrality > 0.35 (Dataset S5).

### Kinetic analysis

Full‐length cDNA of *4CL* genes was subcloned into pET32a(+) vector (Novagen, Schwalbach, Germany) using *EcoR*V/*Bam*HI restriction sites for 4CL1, *Nco*I/*Xho*I for 4CL2 and *Nco*I/*Hind*III for 4CL3. The sequences of primers are given in Table S3. For protein expression, transformed *E. coli* BL21 (DE3) (Novagen) were incubated with shaking at 37 °C in Luria‐Bertani medium containing 50 mg/mL kanamycin until the OD (at 600 nm) reached 0.5–1.0, then induced with 0.25 or 0.5 mm isopropyl 1‐thio‐b‐galactopyranoside and grown at 16 °C overnight. The cultures were harvested by centrifugation at 9000 ***g***. Cell pellets were resuspended in lysis buffer (50 mm Tris–HCl [pH 8.0], 500 mm NaCl, 20 mm imidazole, 10% (v/v) glycerol, 1% (v/v) Tween‐20 and 10 mm b‐mercaptoethanol). Cell lysis was carried out by sonication and centrifugation at 100 000 ***g***. Protein purification was performed using a His Spin Trap column following the manufacturer's instructions (GE Healthcare). The purity of the His‐tag fused proteins was examined by 12% (w/v) SDS‐PAGE, and the concentration was determined by Bradford method.

Kinetic analysis was carried out with a 500 μL reaction buffer (100 mm Tris–HCl, pH 7.5, 2.5 mm ATP, 2.5 mm MgCl_2,_ 0.2 mm CoA and 0.2 mm CoA) in 30 °C (Beuerle and Pichersky, 2002). 0.2–0.4 mm substrates (cinnamic acid, *p*‐coumaric acid, caffeic acid, ferulic acid and sinapic acid) were added. Production of CoA esters was measured by UV spectrophotometer. The change in absorbance was monitored at the wavelengths of 311, 333, 345, 346 and 352 nm for production of cinnamoyl‐CoA, *p*‐coumaroyl‐CoA, feruloyl‐CoA, caffeoyl‐CoA and sinapoyl‐CoA. *V*_max_ and *K*_m_ values were determined from Lineweaver–Burk plots, and *k*_cat_ was determined by dividing *V*_max_ by the enzyme concentration.

### Construction and phenotyping of transgenic hairy root lines

A plant RNAi expression pCAMBIA‐1300‐pHANNIBAL vector was applied for RNA inhibition of *4CL* genes (Wesley *et al*., 2001). According to sequence alignment, an appropriate 400‐ to 500‐bp fragment (Table S4) of each *4CL* gene was selected. Two fragments with reverse and complement sequence were cloned into both ends of the Pdk intron of the vector. For each gene, *Nco*I/*Sal*I restriction sites were selected for amplification of sense fragments, and *Kpn*I/*Bam*HI restriction sites were selected for antisense fragments. The sequences of primers are given in Table S3. The PCR product was cloned into pGEM‐T vector, sequenced and then subcloned on either side of the Pdk intron of the pCAMBIA‐1300‐pHANNIBAL vector. The plasmids were separately introduced into *Agrobacterium tumefaciens* C58C1 (pRiA4) strain, and the positive clones were used to transform *I. indigotica* leaf disc explants. The appropriate vectors without the *4CL* fragments were used as controls. The hairy roots appeared 2 weeks after the infection. The rapidly growing hygromycin‐resistant lines with no bacterial contamination were used to establish hairy root lines. After screening of positive clones, each individual hairy root line was cultured and harvested for transcription and metabolic analysis.

Expression level of genes was detected using qRT‐PCR according to the instructions of SYBR Premix Ex Taq kit (TaKaRa, China). Gene‐specific primers were listed in Table S3. Content of lariciresinol was determined by triple quadrupole mass spectrometer (Agilent 6410; Agilent, Santa Clara, CA) equipped with a pump (Agilent 1200 G1311A; Agilent) and an autosampler (Agilent G1329A; Agilent) according to the method described previously (Chen *et al*., 2013).

## Conflict of interest

The authors declare they have no conflict of interest.

## Supporting information


**Figure S1** Postulate lignans biosynthesis pathway in *I. indigotica*.
***Figure S2** Venn diagram summarising the distribution of differently expressed genes (DEGs) in each samples.*
***Figure S3** GO category of DEGs induced by MeJA.*
***Figure S4** KEGG pathway mapping of DEGs induced by MeJA.*
***Figure S5** PLS‐DA S‐plot of the UHPLC/TOF‐MS spectral from control group and induced samples.*
***Figure S6** Metabolic shifts modulated by MeJA in I. indigotica hairy roots detected in negative mode.*
***Figure S7** GO category of DEGs including in cluster 4.*
***Figure S8** The Pearson correlation network based on the abundance profiles of transcripts in cluster 18 and 21.*
***Figure S9** Expression patterns of 4CLs.*
***Figure S10** Purification of 4CL proteins.*
***Figure S11** Neighbor‐Joining phylogenetic analysis of plant 4CLs.*
**Figure S12** Sequence alignment of Ii4CL with Arabidopsis 4CLs.

**Table S1** Number and quality analysis of reads produced by RNA‐seq in each sample.
***Table S2** Distribution of DEGs in each sample.*
***Table S3** Primers used in this study.*
**Table S4** Sequence of fragments used for RNAi silencing of *4CL* genes.

**Dataset S1** Metabolic profiling.

**Dataset S2** Series clusters analysis for transcriptional profiling.

**Dataset S3** List and transcription profile of candidate genes for co‐expression network construction.

**Dataset S4** Information of genes involved in co‐expression network construction.

**Dataset S5** Information of nodes included in lignan specific co‐expression network.
