# Supplementary material for: Dynamic metabolic and transcriptomic profiling of methyl jasmonate‐treated hairy roots reveals synthetic characters and regulators of lignan biosynthesis in Isatis indigotica Fort
Source: Plant Biotechnol J. 2016 Jun 23;14(12):2217–27. doi: 10.1111/pbi.12576 (PMC5103230; doi:10.1111/pbi.12576)
Supplement: Supplementary file 1 — Figure S1 Postulate lignans biosynthesis pathway in I. indigotica. Figure S2 Venn diagram summarising the distribution of differently expressed genes (DEGs) in each samples. Figure S3 GO category of DEGs induced by MeJA. Figure S4 KEGG pathway mapping of DEGs induced by MeJA. Figure S5 PLS‐DA S‐plot of the UHPLC/TOF‐MS spectral from control group and induced samples. Figure S6 Metabolic shifts modulated by MeJA in I. indigotica hairy roots detected in negative mode. Figure S7 GO category of DEGs including in cluster 4. Figure S8 The Pearson correlation network based on the abundance profiles of transcripts in cluster 18 and 21. Figure S9 Expression patterns of 4CLs. Figure S10 Purification of 4CL proteins. Figure S11 Neighbor‐Joining phylogenetic analysis of plant 4CLs. Figure S12 Sequence alignment of Ii4CL with Arabidopsis 4CLs. [file PBI-14-2217-s007.docx]

**
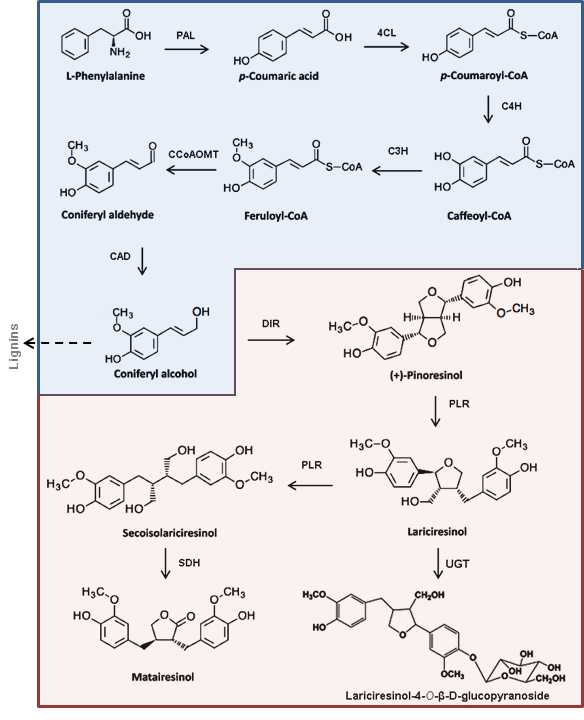
**

**Figure S1**. **Postulate lignans biosynthesis pathway in *I. indigotica*.** Common pathway was marked by blue background, and lignan branch was marked by red background. Abbreviations: 4CL, 4-coumarate: CoA ligase; C4H, Cinnamate; 4-hydroxylase; CCoAoMT, Caffeoyl-CoA O-methyltransferase; DIR, Dirgent; PAL, Phenylalanine ammonia-lyase; PLR, Pinoresinol/lariciresinol reductase; SDH, Secoisolariciresinol dehydrogenase; UGT, UDP-glucose dependent glucosyltransferase.


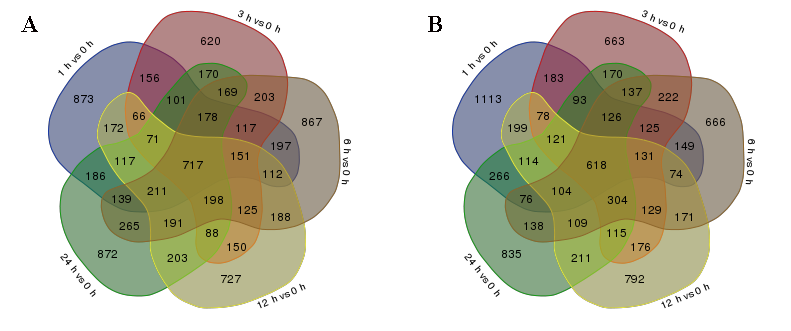


**Figure S2**. **Venn diagram summarising the distribution of differently expressed genes (DEGs) in each samples**. **(a)**. Intersection and unique DEGs of each sample in sample group 1. **(b)** Intersection and unique DEGs of each sample in sample group 2. Venn diagrams were calculated and draw using an on-line analysis tool (<http://bioinformatics.psb.ugent.be/webtools/Venn/>).


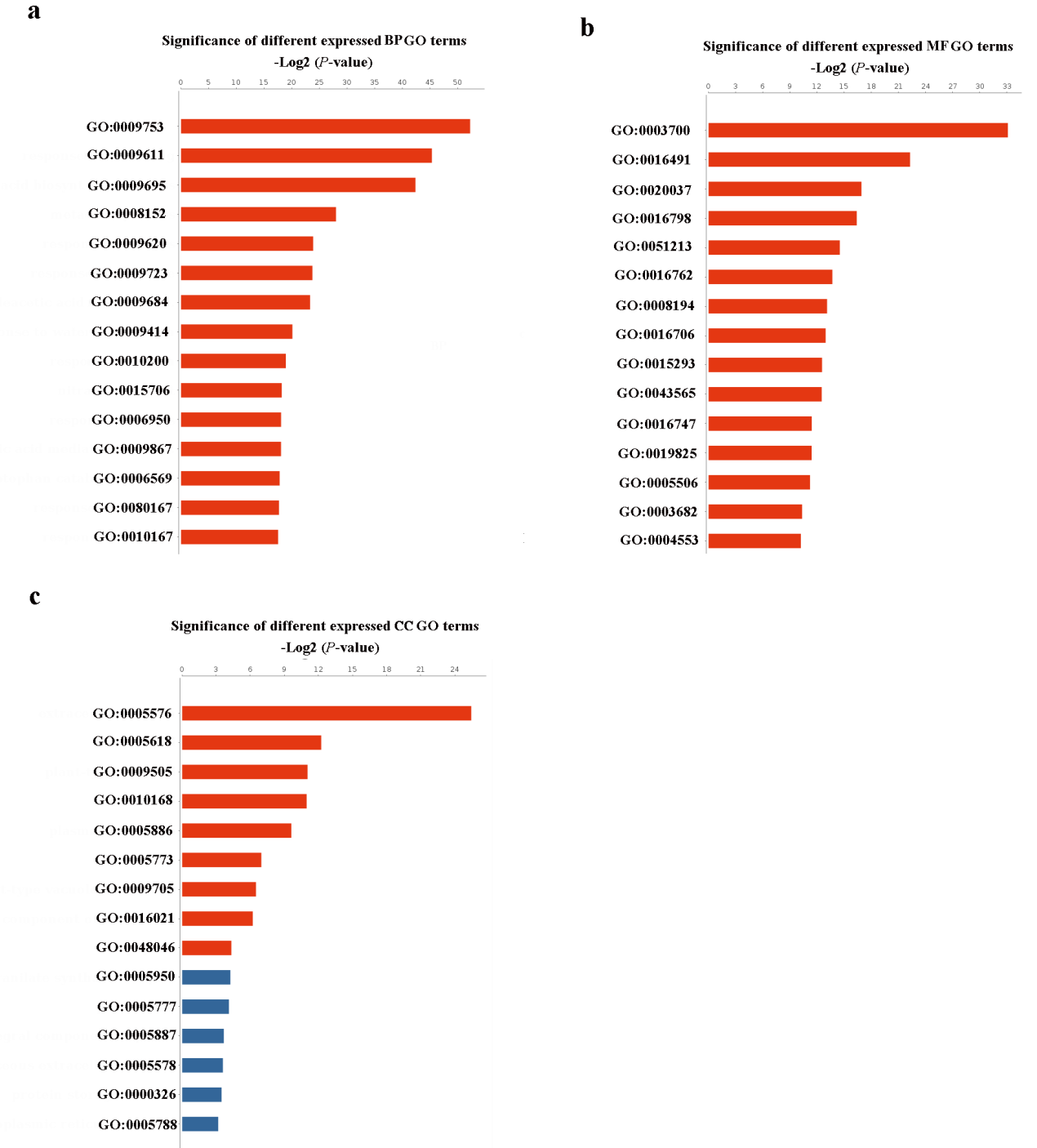


**Figure S3**. **GO category distributions for different expressed genes induced by MeJA**. **(a)**, distribution of GO terms in biological process category (BP). **(b)**, distribution of GO terms in biological molecular function category (MF). **(c)**, distribution of GO terms in biological cellular component category (CC). Bars in red showed GO terms with significant transcriptional changes, blue bars indicated GO terms without significant transcriptional changes. Annotation of GO terms number is as follow. GO:0000326, protein storage vacuole; GO:0003682, chromatin binding; GO:0003700, sequence-specific DNA binding transcription factor activity; GO:0004553, hydrolase activity; GO:0005506, iron ion binding; GO:0005576, extracellular region; GO:0005578, proteinaceous extra cellular matrix; GO:0005618, cell wal; GO:0005773, vacuole; GO:0005777, peroxisome; GO:0005788, endoplasmic reticulum lumen; GO:0005886, plasma membrane; GO:0005887, integral component of plasma membrane; GO:0005950, anthranilate synthase complex; GO:0006569, tryptophan catabolic process; GO:0006950, response to stress; GO:0008152, metabolic process; GO:0008194, UDP-glycosyltransferase activity; GO:0009414, response to water deprivation; GO:0009505, plant-type cell wall; GO:0009611, response to wounding; GO:0009620, response to fungus; GO:0009684, indoleacetic acid biosynthetic process; GO:0009695, jasmonic acid biosynthetic process; GO:0009705, plant-type vacuole membrane; GO:0009723, response to ethylene; GO:0009753, response to jasmonic acid; GO:0009867, jasmonic acid mediated signaling pathway; GO:0010167, response to nitrate; GO:0010168, ER body; GO:0010200, response to chitin; GO:0015293, symporter activity; GO:0015706, nitrate transport; GO:0016021, integral component of membrane; GO:0016491, oxidoreductase activity; GO:0016706, oxidoreductase activity; GO:0016747, transferase activity; GO:0016762, xyloglucan:xyloglucosyl transferase activity; GO:0016798, hydrolase activity; GO:0019825, oxygen binding; GO:0020037, heme binding; GO:0043565, sequence-specific DNA binding; GO:0048046, apoplast; GO:0051213, dioxygenase activity; GO:0080167, response to karrikin.


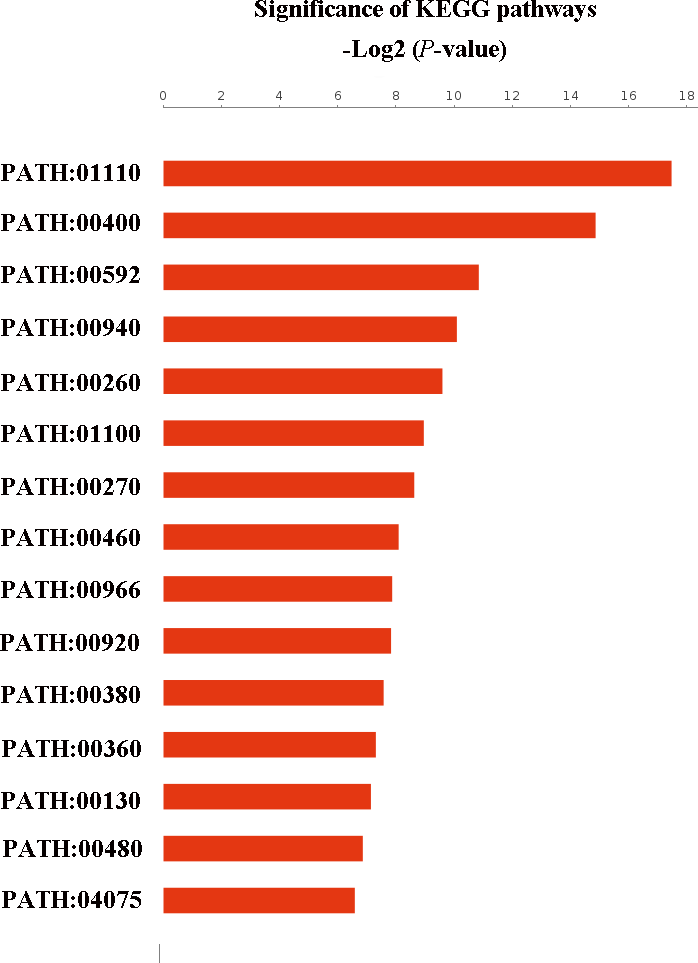


**Figure S4** **KEGG pathway mapping of different expressed genes induced by MeJA**. Annotation of KEGG pathway number is as follow. PATH: 01110, Biosynthesis of secondary metabolites; PATH: 00400, Phenylalanine, tyrosine and tryptophan biosynthesis; PATH: 00592, alpha-Linolenic acid metabolism; PATH: 00940, Phenylpropanoid biosynthesis; PATH: 00260, Glycine, serine and threonine metabolism; PATH: 01100, Metabolic pathways; PATH: 00270, Cysteine and methionine metabolism; PATH: 00460, Cyanoamino acid metabolism; PATH: 00966, Glucosinolate biosynthesis; PATH: 00920, Sulfur metabolism; PATH: 00380, Tryptophan metabolism; PATH: 00360, Phenylalanine metabolism; PATH: 00130, Ubiquinone and other terpenoid-quinone biosynthesis; PATH: 00480, Glutathione metabolism; PATH: 04075, Plant hormone signal transduction.


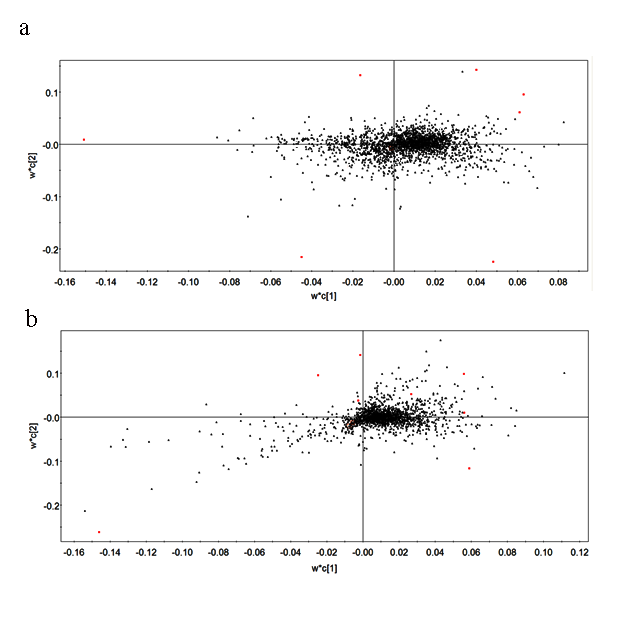


**Figure S5**. **Variable metabolites from** **control group and MeJA induced samples**. **(a)**. Positive mode. **(b)**. Negative mode. Loading plot showed variable metabolites produced by PLS-DA analysis from the LC-MS data sets. Dispersion degree of each plot indicates its degree of variation. Plots marked in red indicated metabolites with VIP value>1 that involved in lignan synthesis.


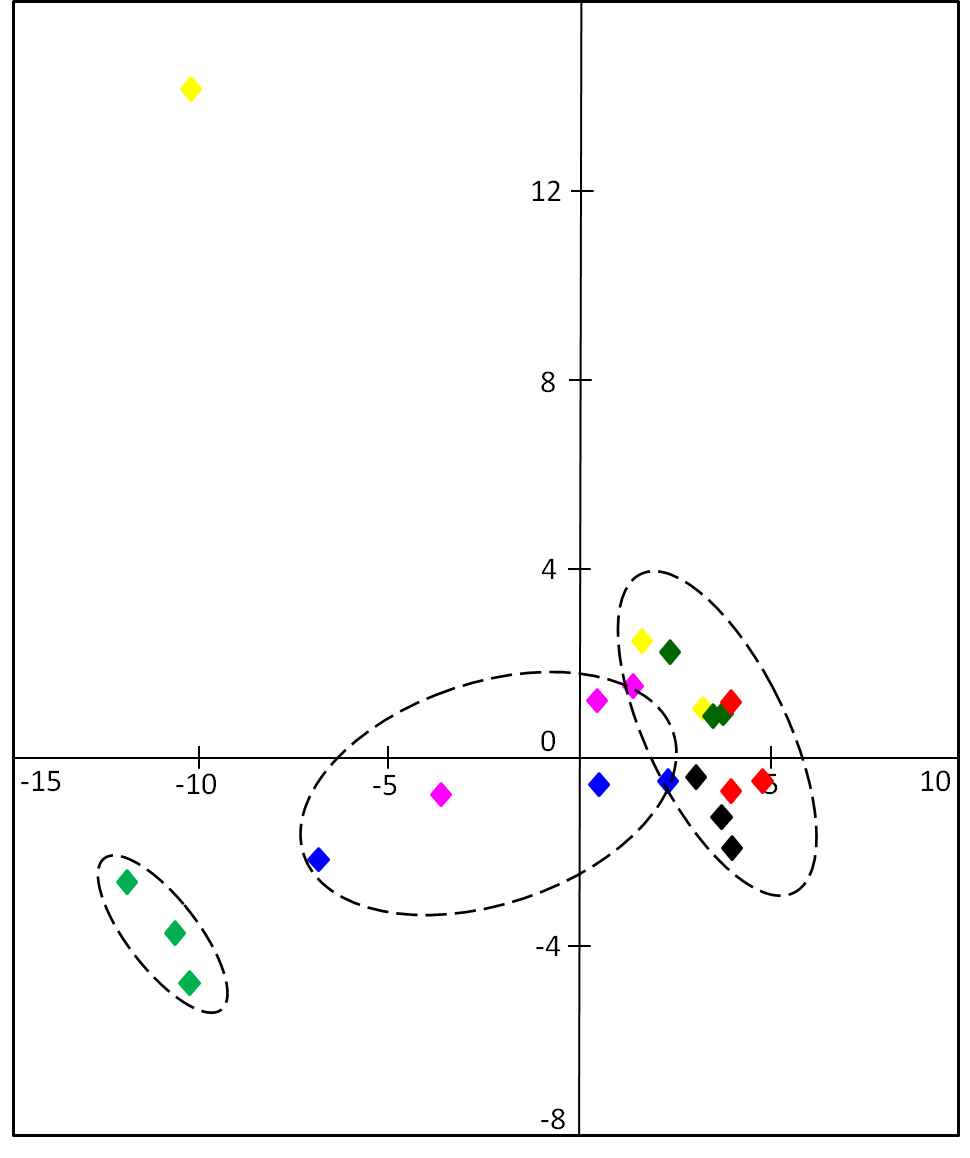


**Figure S6**. **Metabolic shifts modulated by MeJA in *I. indigotica* hairy roots detected in negative mode**. PCA reveals metabolic shifts during the time course of 0-36 h. Plots with different colors indicated samples of different time points. Red, 0 h. Dark green, 1 h. Yellow, 3 h. Pink, 6 h. Black, 12 h. Blue, 24 h. Green, 36 h.


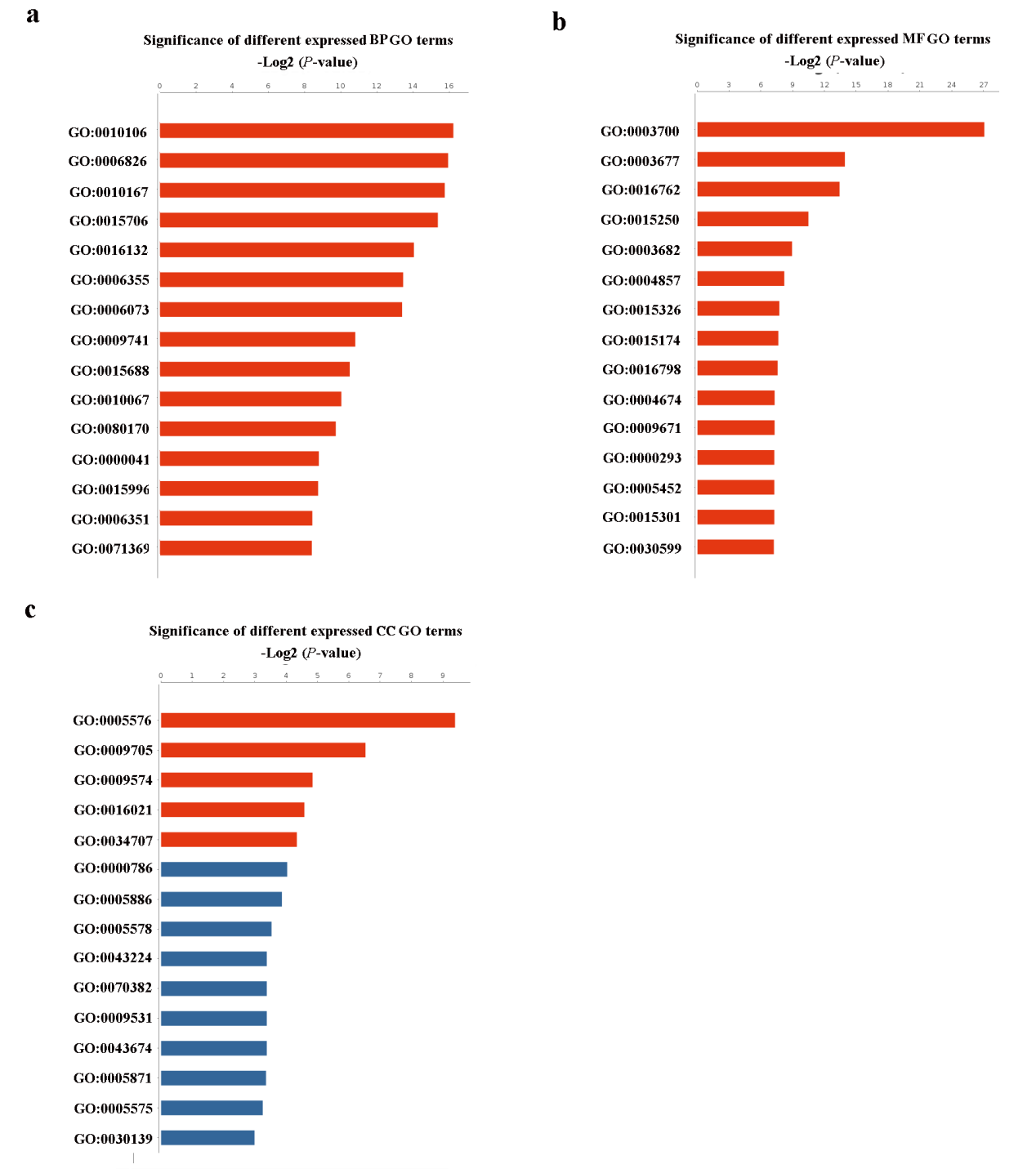


**Figure S7** **GO category contributions for different expressed genes including in cluster4**. **(a)**, distribution of GO terms in biological process category (BP). **(b)**, distribution of GO terms in biological molecular function category (MF). **(c)**, distribution of GO terms in biological cellular component category (CC). Bars in red showed GO terms with significant transcriptional changes, blue bars indicated GO terms without significant transcriptional changes. Annotation of GO terms number is as follow. GO: 0010106, cellular response to iron ion starvation; GO: 0006826, iron ion transport; GO: 0010167, response to nitrate; GO: 0015706, nitrate transport; GO: 0016132, brassinosteroid biosynthetic process; GO: 0006355, regulation of transcription, DNA-templated; GO: 0006073, cellular glucan metabolic process; GO: 0009741, response to brassinosteroid; GO: 0015688, iron chelate transport; GO: 0010067, procambium histogenesis; GO: 0080170, hydrogen peroxide transmembrane transport; GO: 0000041, transition metal ion transport; GO: 0015996, chlorophyll catabolic process; GO: 0006351, transcription, DNA-templated; GO: 0071369, cellular response to ethylene stimulus; GO: 0003700, sequence-specific DNA binding transcription factor activity; GO: 0003677, DNA binding; GO: 0016762, xyloglucan:xyloglucosyl transferase activity; GO: 0015250, water channel activity; GO: 0003682, chromatin binding; GO: 0004857, enzyme inhibitor activity; GO: 0015326, cationic amino acid transmembrane transporter activity; GO: 0015174, basic amino acid transmembrane transporter activity; GO: 0016798, hydrolase activity, acting on glycosyl bonds; GO: 0004674, protein serine/threonine kinase activity; GO: 0009671, nitrate:hydrogen symporter activity; GO: 0000293, ferric-chelate reductase activity; GO: 0005452, inorganic anion exchanger activity; GO: 0015301, anion:anion antiporter activity; GO: 0030599, pectinesterase activity; GO: 0005576, extracellular region; GO: 0009705, plant-type vacuole membrane; GO: 0009574, preprophase band; GO: 0016021, integral component of membrane; GO: 0034707, chloride channel complex; GO: 0000786, nucleosome; GO: 0005886, plasma membrane; GO: 0005578, proteinaceous extracellular matrix; GO: 0043224, nuclear SCF ubiquitin ligase complex; GO: 0070382, exocytic vesicle; GO: 0009531, secondary cell wall; GO: 0043674, columella; GO: 0005871, kinesin complex ; GO:0005575, cellular_component; GO:0030139 , endocytic vesicle.


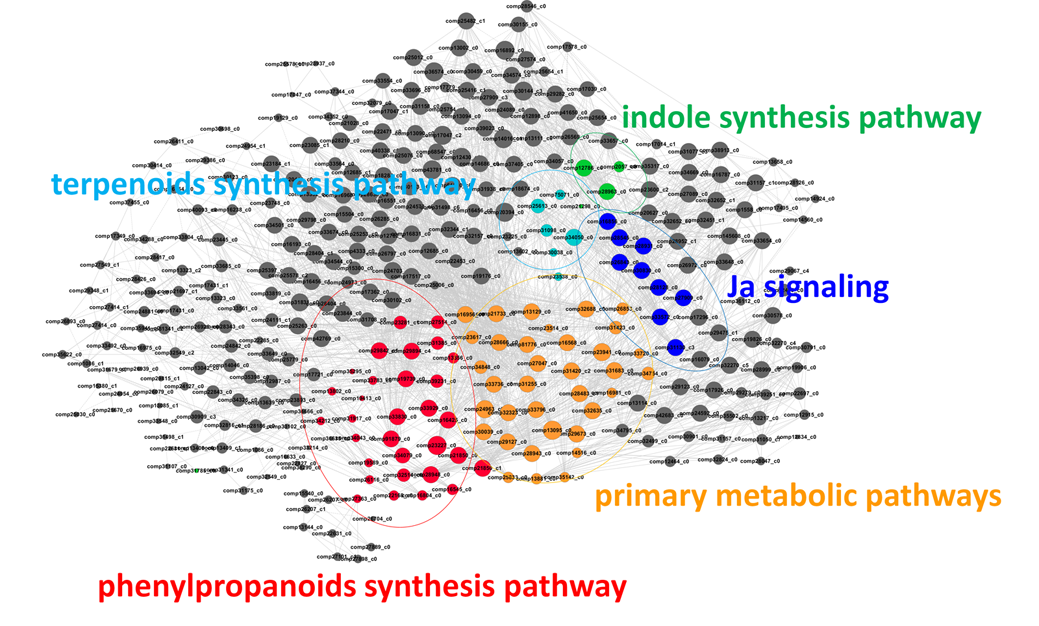


**Figure S8**. **The Pearson correlation network based on the abundance profiles of transcripts in cluster 18 and 21.** The network is visualized with the organic layout in Cytoscape (Shannon et al., 2003). Each node (dot) represents a gene. Size of each node indicates the K-core value of the gene over the network. Each edge (line) represents a positive or negative correlation between the linked pair of nodes. Highly interconnected groups of nodes are located in the same region. Nodes with the same color belong to the same metabolic pathways.


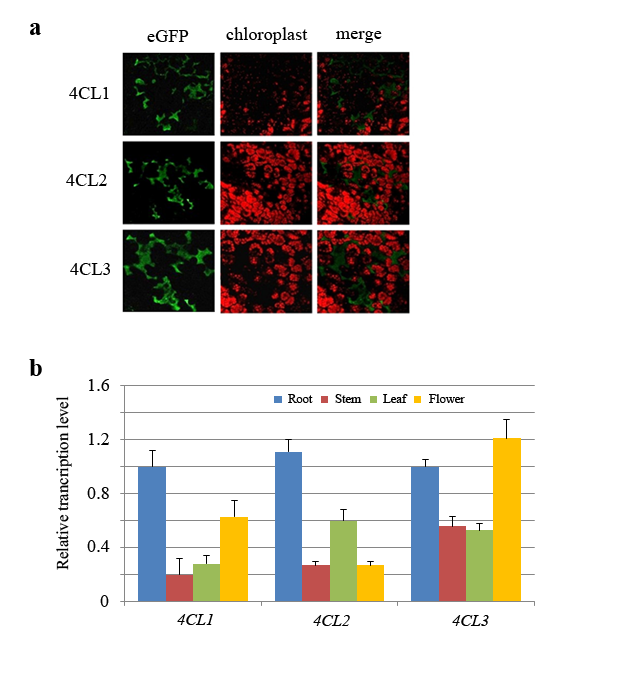


**Figure S9**. **Expression patterns of 4CL genes.** **(a)** To investigate the subcellular localization of 4CLs, each 4CL gene was *C*-terminal fused to the green fluorescent protein (GFP) in the pCAMBIA1300 vector. The resulting construct was transiently expressed in *N. benthamiana* leaves. Confocal microscopy analysis showed all 4CL-GFP fusion proteins had a cytosolic expression pattern. **(b)** Transcription level of each *4CL* gene in different organs of *I. indigotica*. Three *4CL* genes showed similar expression pattern with high transcription level in roots and low level in stems and leaves.


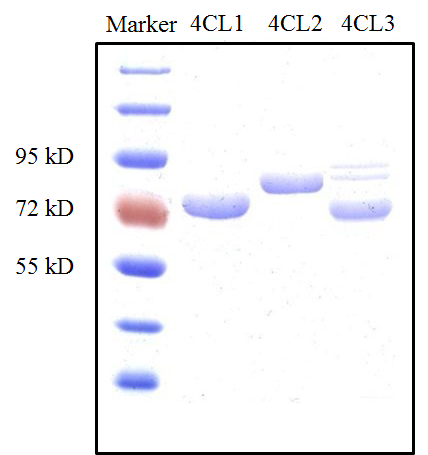


**Figure S10.** **Purification of three recombined 4CL proteins**. Each 4CL protein was expressed in *E. coli* BL21(DE3) and purified. Proteins gel electrophoresis showed each purified 4CL protein with calculated molecular weight of 78 kD, 80 kD, and 77 kD, respectively.


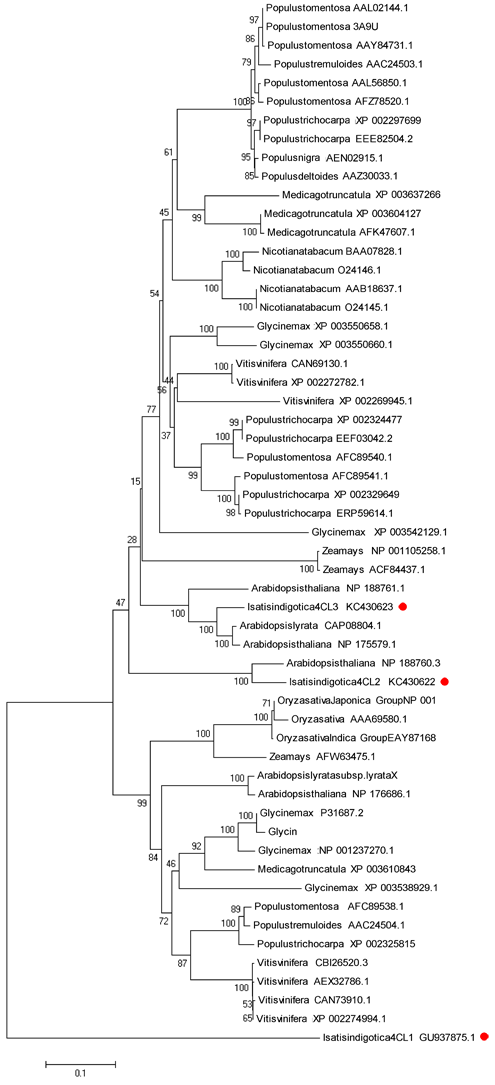


**Figure S11**. **Neighbor-Joining phylogenetic analysis of plant 4CLs**. The branch lengths are proportional to distances, and the values at the interior nodes are the bootstrap percentages derived from 1000 replicates. Accession numbers of the sequences are listed as follow. Followed each species name, the accession number is given. 4CLs from *I. indigotica* were marked by red dots.


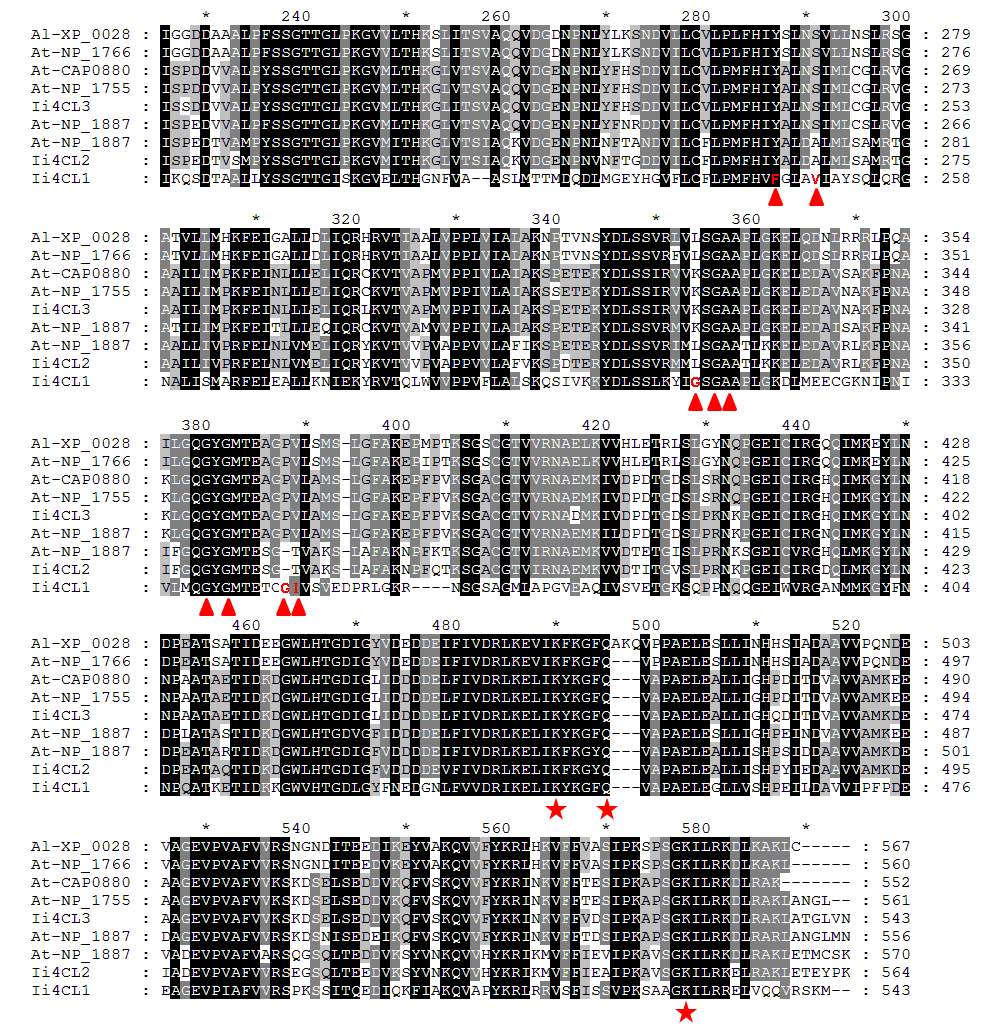


**Figure S12**. **Sequence alignment of Ii4CL with *Arabidopsis* 4CLs.** Residues involved in hydroxycinnamate binding are indicated by triangles, while those involved in enzymatic function are marked by stars. Li4CL1 represented three strictly conserved residues (Lys-444, Glu-449, and Lys-529), which are identified as the catalytic center of 4CLs and indicate a typical 4CL protein. Residues essential for substrates binding (Phe-245, Val-249, Gly-312, Gly-314, Ala-315, Gly-346, and Ile-347) of Ii4CL1 showed obviously difference to other 4CLs.
