# Supplementary material for: Dynamic metabolic and transcriptomic profiling of methyl jasmonate‐treated hairy roots reveals synthetic characters and regulators of lignan biosynthesis in Isatis indigotica Fort
Source: Plant Biotechnol J. 2016 Jun 23;14(12):2217–27. doi: 10.1111/pbi.12576 (PMC5103230; doi:10.1111/pbi.12576)
Supplement: Supplementary file 2 — Table S1 Number and quality analysis of reads produced by RNA‐seq in each sample. Table S2 Distribution of DEGs in each sample. Table S3 Primers used in this study. Table S4 Sequence of fragments used for RNAi silencing of 4CL genes. [file PBI-14-2217-s006.docx]

**Table S1** Numbers and quality of RNA-seq reads produced in each sample. Sample 1 and Sample 2 indicated two biological duplications.

| Samlpe name | Number of reads | Number of base | Q20 ratio (%) |
| --- | --- | --- | --- |
| Sample_1_0h | 35820918 | 3540562541 | 99.34 |
| Sample_1_1h | 43630676 | 4303641236 | 99.31 |
| Sample_1_3h | 39847626 | 3936749044 | 99.32 |
| Sample_1_6h | 53335032 | 5260944877 | 99.32 |
| Sample_1_12h | 43743648 | 4315573504 | 99.31 |
| Sample_1_24h | 39966146 | 3944631761 | 99.32 |
| Sample_2_0h | 42643038 | 4208077724 | 99.30 |
| Sample_2_1h | 51434108 | 5066590330 | 99.25 |
| Sample_2_3h | 34903232 | 3440712289 | 99.26 |
| Sample_2_6h | 39946480 | 3936321687 | 99.25 |
| Sample_2_12h | 43141610 | 4251183197 | 99.25 |
| Sample_2_24h | 48388684 | 4764125639 | 99.21 |

**Table S2** Distribution of DEGs in MeJA induced *I. indigotica* hairy roots.

|  | Sample 1 | | Sample 2 | | Average | |
| --- | --- | --- | --- | --- | --- | --- |
|  | Number of DEGs | Ratio (%) | Number of DEGs | Ratio (%) | Number of DEGs | Ratio (%) |
| 1h VS 0h | 1369 | 2.13 | 1477 | 2.15 | 1423 | 2.14 |
| 3h VS 0h | 1103 | 1.77 | 1125 | 2.02 | 1114 | 1.89 |
| 6h VS 0h | 1473 | 2.30 | 1693 | 2.46 | 1583 | 2.38 |
| 12h VS 0h | 1386 | 2.02 | 1120 | 1.69 | 1253 | 1.86 |
| 24h VS 0h | 966 | 2.52 | 1002 | 1.65 | 984 | 2.09 |

**Table S3** Primers used in this study.

| Primer name | Sequence (5’-3’) | Usages |
| --- | --- | --- |
| Ii4CL2-RealtimeF | cgagatgaacgagcgcg | qRT-PCR |
| Ii4CL2-RealtimeR | gtagcaatggtggcctg | qRT-PCR |
| Ii4CL3-RealtimeF | actccatccaaggttca | qRT-PCR |
| Ii4CL3-RealtimeR | gccggagtagagcttgt | qRT-PCR |
| Actin-F | ccagtggtcgtacaaccggta | qRT-PCR |
| Actin-R | tagttcttttcgatggaggagc | qRT-PCR |
| li4CL1-pC1300-YFP-F(KpnI) | atggtaccatggagaaatccggctacgg | Sub-cellular location |
| Ii4CL1-pC1300-YFP-R(SpeI) | cgactagtcatcttggatcttacttgctg | Sub-cellular location |
| Ii4CL2-pC1300-YFP-F(KpnI) | ccggtaccatgcagaaacagagcagcaa | Sub-cellular location |
| Ii4CL2-pC1300-YFP-R(SpeI) | cgactagtgttcaccaacccagttgcta | Sub-cellular location |
| Ii4CL3-pC1300-YFP-F(KpnI) | gcggtaccatggtgctccaacaacaaca | Sub-cellular location |
| Ii4CL3-pC1300-YFP-R(SpeI) | gcactagtcttagggtactcggtttcca | Sub-cellular location |
| Ii 4CL1-F-(EcoRV ) | gccgatatcatggagaaatccggctacgg | Recombined protein |
| Ii 4CL1-R-(BamHI ) | cgcggatcctcacatcttggatcttactt | Recombined protein |
| Ii4CL2- F-(NcoI) | agccatggcaatggtgctccaacaacaaca | Recombined protein |
| Ii4CL2 –R-(XhoI) | agctcgagtctacttagggtactcggttt | Recombined protein |
| Ii4CL3 -F-(NcoI) | gcccatggctatgcagaaacagagcagcaaca | Recombined protein |
| Ii4CL3 –R-(HindIII) | ggaagcttttagttcaccaacccagttgcta | Recombined protein |
| Ii 4CL1-1304-F (NcoI) | gcccatggcctctttacacgacgaacg | RNAi |
| Ii 4CL1-1304-R (SalI) | gcgtcgactggagagcaaagcattccc | RNAi |
| Ii 4CL1-1304-F-anti (KpnI) | ggggtaccggagagcaaagcattccc | RNAi |
| Ii 4CL1-1304-R-anti (BamHI) | cgggatcccctctttacacgacgaacg | RNAi |
| Ii4CL2- 1304-F (NcoI) | gcccatggtccgtactatacccaatcgg | RNAi |
| Ii4CL2 –1304-R(SalI) | gcgtcgaccgatcaagatcgccgcaccg | RNAi |
| Ii 4CL2-1304-F-anti (KpnI) | ggggtacccgatcaagatcgccgcaccg a | RNAi |
| Ii 4CL2-1304-R-anti (BamHI) | cgggatcctccgtactatacccaatcgg | RNAi |
| Ii4CL3 -1304-F (NcoI) | gcccatggcccttttttcactccggcgg | RNAi |
| Ii4CL3 –1304-R(SalI) | gcgtcgacttatcagaatggccgcaccg | RNAi |
| Ii 4CL3-1304-F-anti (KpnI) | ggggtacc ttatcagaatggccgcaccg | RNAi |
| Ii 4CL3-1304-R-anti (BamHI) | cgggatcc cccttttttcactccggcgg | RNAi |

**Table S4** Sequence of fragments used for RNAi silencing of *4CL* genes.

| Fragment for RNAi silencing of *4CL1* |
| --- |
| cctctttacacgacgaacgaggtttcgaagcagatcaaggactcgaatccgaagcttatcgtctccgttgagcggttattcgacaaagtcaagggctttaatctccccgtcgtgctgctcggttctggcgagtccgtacagattcccgaatcggattcaaaaatcctcactttcgacaacgtgatggagctctccgacccggtttcggatctcccggtcgtcgacatcaagcagtcggacacagctgcgctgttgtattcgtcaggaactacgggaataagcaaaggtgttgaattgactcatgggaacttcgtcgcagcgtctctgatgacgacgatggatcaagatctcatgggagagtatcacggcgtgttcttgtgttttcttcccatgtttcatgtgtttgggcttgccgtgattgcgtattcacagcttcagagagggaatgctttgatctcca |
| Fragment for RNAi silencing of *4CL2* |
| tccgtactatacccaatcggagatcgcaaaacaggcaatagcctccgacgccaagatgatcatcacgaaacggtgttacgtcgataaactaacaaacctccagaacgacggcgtcctgatcgtttgcgtagacgacgaaggcgacgttgtttcgtcagacgacggttgcgtgagtttcacagaactgactaaagcggacgaaacagagcttcctaaaccggaaatctcgccggaggacactgtgtcgatgccgtactcttccggcacaacggggcttccgaagggagtgatgatcactcacaagggattagttacgagcattgctcagaaagtcgacggagaaaaccctaacgtcaacttcaccggagatgacgtcatcctctgttttctcccgatgtttcacatttacgcgctcgacgcgctgatgctctcggctatgaggaccggtgcggcgatcttgatc |
| Fragment for RNAi silencing of *4CL3* |
| cccttttttcactccggcggagatcgcgaaacaggccaaggcctcgaactcgaagctcatcgtcaccgagtctcgctacgtcgacaagatcaaagacctccagaacgacggcgtcatcatcgtctgcaccgacgacgaaccttccccgatcccggaaggctgcctccggttctccgagctgactcagtcaacggaaatcgaaacggtggagatctcttccgacgacgtggtggctcttccttactcctccgggacgacgggtctaccaaaaggagtgatgctgactcacaagggactcatcacgagcgtcgctcagcaagtcgacggcgagaatccgaatctgtacttccacagcgatgacgtcatactctgcgttttgccgatgttccacatctacgctctcaactcgatcatgctgtgtgggcttagagtcggtgcggccattctgataa |
